# Evaluation of flexural strength and antibacterial effect of orthodontic acrylic resins containing Galla chinensis extract

**DOI:** 10.1590/2177-6709.25.6.043-048.oar

**Published:** 2020

**Authors:** Shabnam Ajami, Raha Habibagahi, Reza Khashei, Malihe Soroorian

**Affiliations:** 1 Shiraz University of Medical Sciences, Orthodontic Research Center, Dental School (Shiraz, Iran).; 2 Shiraz University of Medical Sciences, School of Medicine, Department of Bacteriology and Virology (Shiraz, Iran).

**Keywords:** Acrylic resins, Antibacterial, Flexural strength, Natural cariogenic agent

## Abstract

**Objective:**

To evaluate different concentrations of *Galla chinensis* extract (GCE) added to poly(methyl methacrylate) (PMMA), which is widely used for fabrication of removable orthodontic appliances, regarding the effectiveness of this herbal extract on antimicrobial effect and flexural strength of PMMA.

**Methods:**

Acrylic resin samples containing 0.4%, 0.8% and 1.6% GCE were prepared. Flexural strength was investigated via three-point flexural strength test for the 15 acrylic resin blocks of each concentration. Disk diffusion test was used to evaluate antibacterial effects of incorporating the same concentrations of GCE into acrylic resin. All these three groups were compared with the control group, with no added GCE, regarding flexural strength and antibacterial properties.

**Results:**

Comparison of flexural strength between the three study groups and the control group showed significant differences between the groups (P=0.018). However, there was no significant difference between the groups containing GCE. There were significant differences in antimicrobial activity between the four groups (P=0.026).

**Conclusion:**

Within the limitations of this study, it is suggested that incorporation of GCE into PMMA would be beneficial for antimicrobial activity and flexural strength of PMMA, but further studies on other physical properties and antimicrobial effects on other bacterial strain would be beneficial prior to clinical investigations.

## RESUMO

**Objetivo:**

Avaliar se diferentes concentrações de extrato de *Galla chinensis* (EGC) adicionado ao polimetilmetacrilato (PMMA), que é amplamente utilizado para a fabricação de aparelhos ortodônticos removíveis, interferem no efeito antimicrobiano desse extrato e na resistência à flexão do PMMA.

**Métodos:**

Foram preparadas amostras de resina acrílica com concentrações de 0,4%, 0,8% e 1,6% de EGC. Para a avaliação da resistência à flexão, utilizou-se o teste de flexão em três pontos para as 15 amostras de resina em cada concentração. O teste de disco-difusão foi utilizado para avaliar os efeitos antibacterianos da incorporação das mesmas concentrações de EGC na resina acrílica. Esses três grupos foram comparados ao grupo controle, sem adição do EGC, em relação à resistência à flexão e quanto às propriedades antimicrobianas.

**Resultados:**

As comparações dos três grupos com o grupo controle mostraram diferenças significativas (*p*=0,018) para a resistência à flexão. Entretanto, não houve diferença significativa entre os grupos contendo EGC. Foram encontradas diferenças significativas na atividade antimicrobiana entre os quatro grupos (*p*=0,026).

**Conclusão:**

Dentro das limitações desse estudo, parece que a incorporação de EGC no PMMA seria benéfica para a atividade antimicrobiana e a resistência à flexão do PMMA. Porém, estudos adicionais sobre outras propriedades físicas e sobre os efeitos antimicrobianos contra diferentes cepas de bactérias seriam interessantes antes de se fazer pesquisas clínicas.

## INTRODUCTION

Acrylic orthodontic appliances can create great biofilm accumulation on dental surfaces and retentive sites of acrylic baseplate, making it a challenge for patients to maintain adequate oral hygiene especially in bonded appliances. Orthodontic appliances can increase the levels of mutans streptococci (MS) in saliva and dental biofilm during active removable orthodontic treatment. Therefore, dental caries commonly occur in areas adjacent to the irregular orthodontic appliance surfaces.^1^ ^,^ ^2^ Batoni et al.^3^ evaluated the effect of removable orthodontic appliances on oral colonization by mutans streptococci in children, and showed that the use of removable acrylic appliances may lead to the creation of new retentive areas and surfaces, which favors the local adherence and growth of MS. Although acrylic resins (AR) are extensively used for fabricating removable orthodontic appliances, including retainers, functional appliances and even bonded orthodontic appliances, accumulation of plaque is one of the major drawbacks.^4^ ^-^ ^6^ Their surface porosities have potential for retention of food, attribute the increase activities of cariogenic microorganisms in the oral cavity.^7^ It is absolutely necessary to develop strategies in order to effectively prevent enamel demineralization during application of acrylic appliances as the mechanotherapies during orthodontic treatments. Use of various antibacterial materials in the fabrication of orthodontic appliances and adhesive resins is a new area for investigation in orthodontics.^8^ ^,^ ^9^ New antibacterial agents might be effective in preventing demineralization of enamel through inhibition of colonization and proliferation of cariogenic bacteria, since they are the primary etiologic agents for the development of white spot lesions.^7^ ^,^ ^10^ ^,^ ^11^

Novel medicines derived from natural products help investigators to discover new materials with anticariogenic properties.^12^ *Galla chinensis* extracts (GCE), a traditional Chinese medicine, is an effective anticariogenic agent, which inhibits the growth and metabolism of cariogenic bacteria.^13^ ^-^ ^15^ Demineralization/remineralization balance of the enamel might be favorable in the presence of GCE.^16^ Furthermore, recent studies have shown that GCE inhibits the proliferation and production of acids by cariogenic bacteria, including *Streptococcus mutans* and *Lactobacillus rhamnosus*.^17^ In 2008, a study on the multi-species biofilm models showed higher pH levels, lower counts of cariogenic bacteria and less compact biofilms in flow cells with *Galla chinensis* extracts.^18^ In addition as a positive character, another study did not show any change in the bond strength of adhesive cements containing GCE.^9^ Apart from bacterial activity, the mechanical properties of acrylic resins are equally important; in this context, flexural strength (Fs) is one of the important physical properties that should be evaluated, especially in acrylic appliances. A standard minimum limit has been defined for the flexural strength of acrylic resin types by ISO 20795-1 (2008) for dental base polymers. The flexural strengths of polymerized materials should not be <50 MPa.^19^ Therefore, researchers strongly recommended that the effects of additives or modifiers on the mechanical properties of acrylic materials be evaluated to avoid detrimental effects that might decrease their strength to values lower than the standard value. This study was undertaken to evaluate the effect of incorporation of different concentrations of *Galla chinensis* as a phytochemical antibacterial component into poly(methyl methacrylate) (PMMA) on the antibacterial properties without deteriorating the physical properties of this material, by investigating the flexural strength of the material with different concentrations of GCE.

## MATERIAL AND METHODS

### Preparation of GCE-containing polyacrylic discs

For preparation of all the discs with the same size, a mold was designed, measuring 9 mm in diameter and 1mm in thickness, based on Neo-Sensitabs Tablets (Rosco Diagnostica, Denmark). The GCE powder was added to the liquid of PMMA in proposed fractions, to achieve the following mass fraction of GCE in PMMA mixtures: 0% (control group), 0.4%, 0.8% and 1.6%. To prepare the concentrations mentioned above, 0.032 g, 0.064 g and 0.128 g of GCE were added to each mL of acrylic monomer, respectively. Five samples were prepared for each fraction of GCE impregnation.

### Preparation of blood agar (tryptone soy agar with 5% blood)

This culture medium was used as a primary environment for the culture and purification of bacteria. Forty grams of medium powder were dissolved in 1 liter of distilled water. Then the medium was put in the autoclave at a 121^o^C temperature and 15 psi pressure, for 15 minutes. Then the medium was put at room temperature to cool down. At this time, 5% of defibrinated blood was added to the culture medium under wholly sterile conditions and covered, to prevent medium from outside contamination.

### Preparation of the Muller-Hinton agar medium

This medium was used for antibiogram testing. Thirty-eight grams of medium powder were solved in one liter of distilled water and then sterilized by autoclave. In sterile conditions, the medium was poured into sterile plates. The thickness of the environment was 4mm (about 30mL per plate).

### Diffusion test

Disk diffusion technique was applied to evaluate the antibacterial effect. *Streptococcus mutans* suspension was inoculated on four plates with at least 20mL of Mueller-Hinton agar (MHA) with 5% sheep blood. Five discs were loaded on each plate. The plates were incubated at 37°C with 5% CO_2_. A digital caliper was used to measure the inhibition halo diameters after 24h of incubation. The measurements mentioned above were repeated three times and the mean value was subjected to statistical analysis.

### Three-point flexural strength test

PMMA acrylic resin block samples for each different fraction of GCE were prepared as follows. The dimension of the constructed block for 3-point flexural test was 30×5×2 mm. The test was carried out in the four study groups, each containing 15 specimens, with different concentrations of the GCE. Acrylic resin powder containing 0%, 0.04%, 0.8% and 1.6% GCE was mixed with monomer at 25°C; all the procedural steps were carried out by one operator. The mixture was transferred into a silicon mold in its doughy stage during polymerization. After completion of the settling of acrylic specimens, favorable dimensions were achieved by a grinding procedure in the turnery. Before carrying out the flexural strength tests, the prepared specimens were immersed in 37°C distilled water for two weeks, to simulate the oral environment. A universal testing machine (Zwick Z020 Germany) was used for the 3-point flexural strength test. The surface area of the acrylic resin block was determined, and the load at fracture (N) was recorded. The pre-load force was 0.5 N, followed by a gradual increase at a rate of 0.5 mm/min. The load (N) at fracture was recorded for each sample according to the formula below:

$$ɒ=\text{F}\times \text{L}\times 3/2\times \text{b}\times {\text{h}}^{2}$$

**Figure 1:**
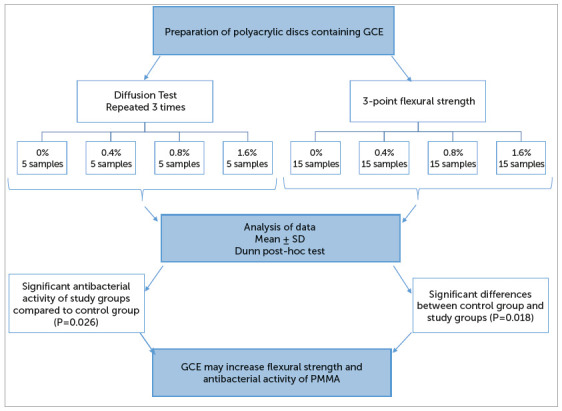
Illustration of methods and materials.

Where **ɒ**= flexural strength, F= the maximum force (N), L = the distance between the supporting arms of the machine (mm), b = the specimen width (mm) measured immediately before storage in water, and h = the specimen height (mm) measured immediately before storage in water. As mentioned before, the values for L, b and h were 30 mm, 5 mm and 2 mm, respectively. An auto-polymerizing acrylic resin (Acropars, Self-cured, Iran) was used in this study. The method sequence is briefly presented in the flowchart (Fig 1).

SBS and antimicrobial activity data were described using median, interquartile range (IQR), means and standard deviations (±SD). The non-parametric Kruskal-Wallis H statistical test and *post-hoc* Dunn test were used to compare the groups. SPSS 22.0 (IBM) was employed for data analysis. Statistical significance was set at *p*<0.05.

Ethical considerations were confirmed by the Ethics Committee of Shiraz University of Medical Sciences (IR.SUMS.REC.1397.804).

## RESULTS

Table 1 presents the comparisons of flexural strengths, indicating significant differences between the control group and the study groups (P=0.018). However, there were no significant differences between groups containing GCE. According to Table 2, there were significant differences in antimicrobial activities between the study groups and the control group (P=0.026).The antimicrobial activity of the acrylic resins containing different percentage of GCE against *Streptococcus mutans* increased after 24 hours. However, no significant trend was observed between the study groups.

**Table 1:** Comparisons of flexural strengths.

Group	mean±SD	Median	IQR^*^	Dunn post-hoc	p-value^**^ between groups
1 (control)	62.95 ± 7.32	63.80 ^A^	11.25		0.018
2 (0.4% GCE)	72.95 ± 7.46	72.10 ^B^	11.25	p=0.007	
3 (0.8% GCE)	72.36 ± 11.33	76.70 ^B^	21.25	p=0.006	
4 (1.6% GCE)	69.51 ± 9.88	72.85 ^B^	18.00	p=0.047	

* inter-quartile range. ** Kruskal-Wallis H test. Median values with at least a common superscript letter were not statistically different (Dunn post-hoc test).

## DISCUSSION

This study was undertaken to investigate the effect of GCE on the flexural strength and antibacterial activity of self-cured polymethyl methacrylate resin (Acropars). The results of this study indicated that incorporation of GCE improved the mechanical and antibacterial activity of PMMA (Tables 1 and 2). Microbial plaque can adhere to the surface of acrylic resin appliances at a wider adhesion area, compared to natural teeth;^20^ mechanical methods proved ineffective in removing microorganisms completely.^21^ ^,^ ^22^ Many researchers have made attempts to develop effective and harmless techniques to incorporate self-sterilizing agents into acrylic resins.^23^ In this context, incorporation of GCE into the matrix of polymeric materials as an antimicrobial agent has attracted much attention in recent years.^13^ ^-^ ^15^ Based on the present results, incorporation of GCE at all the three concentrations resulted in an increase in antibacterial activity, compared to the control group, in accordance with previous studies.^14,15^ In addition, the results showed that the acrylic resin containing GCE had a strong antibacterial effect on *S. mutans*. Further clinical experiments would be useful, especially for different bacterial and fungal components. Apart from the inhibition of enamel demineralization effect observed, in a previous study GCE promoted remineralization of incipient enamel lesions and inhibited metabolism of oral bacteria, suggesting that it might be a potential and promising anticariogenic agent.^17^ Based on FS test, the present findings are different from previous studies, such as a study by Shibata et al.^24^ and Sodagar et al.,^25^ indicating that incorporation of silver nanoparticles (Ag NPs) into acrylic resins would decrease flexural strength, since it serves as impurities, affecting the internal structure of PMMA.^23,26,27^ The study by She^28^ showed that incorporation of Ag Nps into denture base resins resulted in the growth inhibition of *Streptococcus mutans*, with no significant effect on the mechanical properties of the denture base resin. The results of this study, for the first time, showed the positive effect of GCE on the mechanical properties of acrylic resins. Although further studies are necessary, the results could be justified as follows. Acrylic resins commonly consist of methacrylates, especially PMMA, with this chemical formula: (C_5_O_2_H_8_), polyethyl methacrylate and additional copolymers.^4-6^ *Galla chinensis* is also rich in gallotannins (C_5_H_10_N_2_O_3_), with nearly 20% of gallic acid (C_6_H_2_(OH)_3_COOH) and 7% of methyl gallate.^29^ Gallotannins consist of a central glucose core (C_6_H_12_O_6_), which is surrounded by several gallic acid (GA) units, and further GA units can be attached through bonding of additional galloyl residues.^16^ In addition, inorganic ions could in part be responsible for the clinical effects of natural medicines. In the present study, nitrogen ions of the glutamine part were found to produce strong covalent bonds.^30^ Carbon-nitrogen is a covalent bond which is one of the well-known bonds between carbon and nitrogen atoms. Given that nitrogen has three electron capacities and carbon has four electrons, these two atoms can create 1-3 covalent links at a time. In addition, since GCE contains a significant amount of polyphenols, the hydroxyl and carboxyl groups of polyphenols might form several hydrogen bonds with bulky acidic and basic amino-acid side chains in the band region of PMMA. In summary, the chemical interaction between the two substrates increased the flexural strength of the block. An increase in flexural strength might indicate that *Galla chinensis* extract can intercalate the resin chains and strengthen the intermolecular interactions. Since this research was limited to one type of acrylic resin, further studies are recommended for evaluation of the effects of different concentrations in other types of acrylic resin. It should be pointed out that GCE might affect the flexural strength of some types of acrylic resin and therefore the advantages of their antimicrobial properties should be considered *versus* the possible effect on flexural strength or some other physical and mechanical properties. Another limitation of our study was using Agar diffusion test, which was used to evaluate the overall antimicrobial properties of this substance, due to its simplicity and low cost. However, for further evaluation and dilution determination, other minimum inhibitory concentration (MIC) tests should be performed.

**Table 2:** Antimicrobial activities between groups.

Group	Mean ± SD	Median	IQR^*^	p-value^**^
1 (control)	0.0 ± 0.0	0.0 ^A^	0	< 0.001
2 (0.4% GCE)	11.00 ± 1	11.00 ^B^	6.25	
3 (0.8% GCE)	13.66 ± 0.57	14.00 ^BC^	4.50	
4 (1.6% GCE)	15.66 ± 0.57	16.00 ^C^	4.50	

* inter-quartile range. ** Kruskal-Wallis H test. Median values with at least a common superscript letter were not statistically different (Dunn post-hoc test).

## CONCLUSION

Under the limitations of this study, the results suggested that *Galla chinensis* extract might be used as a newly introduced natural cross-linker to stabilize acrylic resins, which improves resistance to flexural strength and bacterial activity. Chemical interaction between GCE and PMMA was the responsible for increasing the flexural strength of the block.
